# Do RATs save lives? A service evaluation of an out-of-hospital cardiac arrest team in an English ambulance service

**DOI:** 10.29045/14784726.2019.03.3.4.32

**Published:** 2019-03-01

**Authors:** Richard Pilbery, M. Dawn Teare, Daniel Lawton

**Affiliations:** Yorkshire Ambulance Service NHS Trust; University of Sheffield; University of Huddersfield; Yorkshire Ambulance Service NHS Trust

**Keywords:** cardiac arrest, out-of-hospital, paramedic

## Abstract

**Introduction:**

Out-of-hospital cardiac arrest (OHCA) is a major public health problem, leading to a substantial number of deaths in the UK. In response to this, the Yorkshire Ambulance Service NHS Trust (YAS) has introduced red arrest teams (RATs). RAT members attend a three-day training course, focusing on the technical and non-technical skills that are required to effectively team lead an OHCA and provide high quality post-resuscitation care. This evaluation aims to determine the impact of the RATs on survival to 30 days and return of spontaneous circulation (ROSC) at hospital.

**Methods:**

All adult (≥ 18 years) OHCAs entered onto the YAS computer aided dispatch (CAD) system between 1 October 2015 and 30 September 2017 were included if the patient was resuscitated and the cause of the arrest was considered to be medical in origin. Multi-variable logistic regression models were created to enable adjustment for common predictors of survival and ROSC.

**Results:**

During the 2-year data collection period, YAS attended 15,151 cardiac arrests. After removing ineligible cases, 5868 cardiac arrests remained. RATs attended 2000/5868 (34.1%) incidents, with each RAT attending a median of 13 cardiac arrests (IQR 7–23, min. 1, max. 78).

The adjusted odds ratios suggest that a RAT on scene is associated with a slight increase in the odds of survival to 30 days (OR 1.01, 95% CI 0.74–1.38) and odds of ROSC on arrival at hospital (OR 1.13, 95% CI 0.99–1.29), compared to the odds of not having a RAT present, although neither result is statistically significant.

**Conclusion:**

The presence of a RAT paramedic was associated with a small increase in survival to 30 days and ROSC on arrival at hospital, although neither were statistically significant. Larger prospective studies are required to determine the effect of roles such as RAT on outcomes from OHCA.

## Introduction

Out-of-hospital cardiac arrest (OHCA) is a major public health problem leading to a substantial number of deaths in Europe. Since routine reporting of cardiac arrest outcomes commenced in the UK in 2011, it is evident that even in the Utstein group of patients (i.e. those who suffered a witnessed cardiac arrest of presumed cardiac cause, were resuscitated and found to be in a shockable rhythm on arrival of the ambulance service), survival to discharge rates have remained under 30%, well below that of the best performing European countries (Gräsner et al., 2016).

In response to this, the Yorkshire Ambulance Service NHS Trust (YAS) has introduced several initiatives to improve the outcome from OHCA, including:

- teaching basic life support (BLS) to members of the public, particularly school-age children;
- improving telephone triage of 999 calls to ensure that there is a minimum delay in recognition of cardiac arrest and commencement of telephone CPR; and
- introducing red arrest teams (RATs) across Yorkshire.

The red arrest teams (RATs) consist of operational managers who attend a three-day training course, focusing on the technical and non-technical skills that are required to effectively team lead an OHCA and provide high quality post-resuscitation care. RATs have been deployed throughout Yorkshire, ensuring that all members of the public can benefit from the initiative, and not just one locality. The RAT scheme is also different from some other services, such as those provided by the London and South East Coast Ambulance Services, in that the training is not at Master’s degree level or associated with a prolonged training period, making it inexpensive and pragmatic to run despite high operational pressures and economic constraints.

Following the introduction of the RAT scheme and other initiatives, an internal audit showed that YAS achieved Utstein survival to discharge rates in excess of 41%, compared to the national average in the same period of 28%, in 2015–2016. However, the relative contribution of each aspect of the initiatives that have been introduced within the service is unknown.

This study aims to determine the impact of the RATs on outcomes from OHCA, comparing patients who were attended by RATs and those who were not. The primary outcome measure is survival to 30 days. The secondary outcome measure is a return of spontaneous circulation (ROSC) on arrival at hospital.

## Methods

A retrospective cohort study analysing routinely collected data between October 2015 and September 2017 was undertaken, to compare differences in outcomes from OHCAs, between incidents where a RAT paramedic was present and incidents where a RAT paramedic was not present. Multiple logistic regression was used to adjust for factors that are known to affect outcomes from OHCA.

### Setting

YAS provides 24-hour emergency and healthcare services for the county of Yorkshire, in England. The county has a population of approximately five million, spread over almost 6000 square miles of varied terrain, including isolated moors and dales, coastline and urban areas. YAS operates 62 ambulance stations, and in 2016–2017 received 895,700 emergency calls which resulted in 723,935 attendances by YAS staff.

### Red arrest team

The RAT concept in YAS started thanks to staff in the Hull area taking the initiative and undertaking the role informally in 2013. The following year, based in part on the work of the Resuscitation Rapid Response Unit (3RU) in Scotland (Clarke, Lyon, Short, Crookston, & Clegg, 2014) and an American Heart Association consensus statement on CPR quality and improving outcomes from cardiac arrest (Meaney et al., 2013), formal pilots were conducted in Bradford, Doncaster, Harrogate, Hull, Leeds and York. RAT members were provided with a one-day training course, with a syllabus focused on team leadership and other non-technical skills, in addition to doing the basics well (e.g. increasing chest compression fraction, providing high-quality ventilation). Following the pilot phase, a widespread roll-out occurred from October 2015 to all existing operational line managers (referred to locally as clinical supervisors). From 2016/2017, the RAT course was extended to three days, to include additional skills such as post-ROSC care, and included an assessment of competence (Supplementary 1). In addition, RATs undergo an annual clinical refresher and re-assessment.

YAS has a pre-determined response to cardiac arrest calls which is comprised of at least two resources, including a conveying resource, i.e. an ambulance, and at least one advanced life support (ALS) provider (paramedic). A RAT paramedic is also dispatched if they are available and are located less than a 20-minute drive from the patient’s location.

### Data collection

Cardiac arrests were identified from the YAS computer aided dispatch (CAD) system via a bespoke database query, and by review of patient care records (PCR) by a research paramedic. Outcome data were obtained from the YAS clinical audit and business intelligence units as part of their routine reporting of ambulance quality indicators (NHS England, 2015). The clinical directorate at YAS provided a list of RAT-trained paramedics, along with the date they completed their training, in addition to the call signs of RAT vehicles. This was cross-referenced against the ambulance staff who had attended a cardiac arrest, to determine if a RAT had attended the incident. When calculating the elapsed time from cardiac arrest to RAT arrival, only the first RAT-trained paramedic’s time was included (i.e. if more than one RAT-trained paramedic was in attendance, subsequent RAT arrival times were ignored). Where the cardiac arrest onset time was not known, the emergency call time was used instead.

In addition to the RAT presence and time of arrival, the age, gender and location of the patient was recorded. Other variables included whether bystander CPR occurred; whether the OHCA was witnessed, and if so, by whom; the response time of the first YAS response; the presenting rhythm; the pre-hospital outcome (i.e. whether the patient was transported to hospital or had a Recognition of Life Extinct (ROLE) performed on scene); and the hospital outcome, consisting of the presence of ROSC on arrival at hospital and the survival outcome.

### Participants

All adult (≥ 18 years) OHCAs entered onto the YAS CAD system between 00:00:00 on 1 October 2015 and 23:59:59 on 30 September 2017 were included if the patient was resuscitated and the cause of the arrest was considered to be medical in origin. Incidents were excluded if the PCR could not be located, resuscitation was not commenced or continued by a member of YAS staff or the cardiac arrest was of traumatic origin or occurred in-hospital. In addition, to account for appropriate termination of futile resuscitations, that form part of the RAT role, all cardiac arrests where resuscitation was terminated within 10 minutes of RAT arrival on scene, or 10 minutes of the first crew arrival time on scene, were excluded.

### Statistical analysis

Multi-variable logistic regression models were created using the statistics package R (R Core Team, 2017), to enable adjustment for known factors that affect OHCA survival and ROSC, including patient age, gender, location, whether the arrest was witnessed and if so by whom (bystander or ambulance crew), whether bystander CPR was performed, response time and first monitored cardiac rhythm. In addition, the presence or absence of a RAT paramedic was noted.

## Results

**Figure 1:**
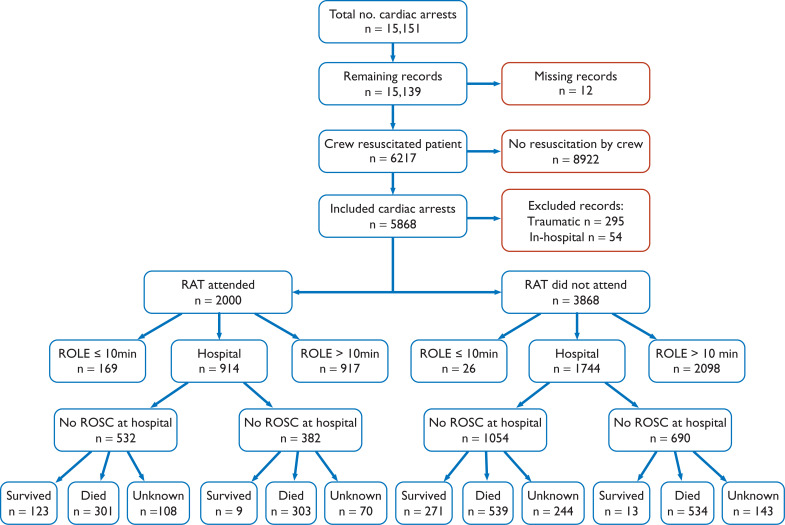
Figure 1. Patients suffering an OHCA in the study period.

Between 1 October 2015 and 30 September 2017, there were 15,151 cardiac arrests that were attended by YAS. After removing 12 cases where no PCR could be located, 15,139 remained. There were 8922 patients who had no resuscitation attempted by YAS ambulance personnel, and 6217 cardiac arrests where resuscitation was attempted. Another 349 were removed since the cardiac arrest was either of traumatic origin (295 incidents), or was an in-hospital cardiac arrest (54 incidents). This resulted in 5868 cardiac arrests suitable for inclusion in this evaluation (Figure 1).

During the 2-year data collection period, 123/158 (77.8%) RATs attended 2000/5868 (34.1%) incidents, with each RAT attending a median of 13 cardiac arrests (IQR 7–23, min. 1, max. 78). The demographics of the two patient groups (RAT/non-RAT) were similar (Table 1), although there were several significant differences in the distribution of patient demographic and OHCA factors between the RAT attended and non-RAT attended OHCAs.

**Table 1.** Demographic details of cardiac arrests.

Variable	RAT	No RAT	All
n (%)	2000 (34.1)	3868 (65.9)	5868 (100)
Median age (IQR) years	70 (57–80)	72 (60–82)	71 (59–81)
Male n (%)	1256 (62.8)	2420 (62.6)	3676 (62.6)
Bystander CPR n (%)	1344 (67.2)	2366 (61.2)	3710 (63.2)
Median response time by crew mins (IQR)	7 (5–10)	7 (4–10)	7 (4–10)
Median response time by RAT mins (IQR)	15 (10–22)	NA (NA)	15 (10–21)
**Witness status n (%)**			
Unwitnessed arrests	806 (40.3)	1433 (37.0)	2239 (38.2)
Witnessed arrests	1194 (59.7)	2435 (63.0)	3629 (61.8)
Witnessed arrest by EMS	129 (6.5)	565 (14.6)	694 (11.8)
Witnessed arrest by bystander	1065 (53.2)	1870 (48.3)	2935 (50.0)
**Presenting rhythm n (%)**			
Shockable	515 (25.8)	997 (25.8)	1512 (25.8)
PEA	402 (20.1)	863 (22.3)	1265 (21.6)
Asystole	1083 (54.1)	2008 (51.9)	3091 (52.7)
**Location n (%)**			
Private	1500 (75)	2822 (73)	4322 (73.7)
Public	329 (16.4)	570 (14.7)	899 (15.3)
Nursing home	162 (8.1)	357 (9.2)	519 (8.8)
Ambulance	9 (0.4)	119 (3.1)	128 (2.2)
**Pre-hospital measure n (%)**			
Resuscitation > 10 mins	917 (45.9)	2098 (54.2)	3015 (51.4)
Resuscitation < 10 mins	169 (8.5)	26 (0.7)	195 (3.3)
Transported to hospital	914 (45.7)	1744 (45.1)	2658 (45.3)
**Hospital/survival measures n (%)**			
ROSC at hospital	532 (26.6)	1054 (27.2)	1586 (27.0)
Survived	132 (6.6)	284 (7.3)	416 (7.1)
Survival status unknown	178 (8.9)	387 (10.0)	565 (9.6)
Died in hospital	604 (30.2)	1073 (27.7)	1677 (28.6)

NA: not applicable.

RATs attended OHCAs with slightly younger patients (median 70 years vs. 72 years) and a higher proportion of bystander-witnessed arrests (53.2% vs. 48.3%), and were more likely to terminate an OHCA within 10 minutes of arriving on scene (8.5 vs. 0.7). Conversely, in the non-RAT OHCA group, there was a significantly higher proportion of witnessed arrests by a member of the ambulance service (14.6% vs. 6.5%), and cardiac arrests that occurred in an ambulance (3.1% vs. 0.4%).

There were 2658 patients who were conveyed to hospital, although the survival outcome for 605 incidents was initially unknown. Clinical audit had not identified 519 incidents as an OHCA, and a further 86 OHCAs that had been identified by clinical audit received no survival outcome data from the destination hospital. However, as a result of screening the CAD and reviewing PRFs, the outcome of a further 40 was determined, although none of these patients survived to discharge. This resulted in a final total of 565/2658 (21.3%) patients with no survival outcome status.

### Roll-out of the RAT scheme

**Figure 2:**
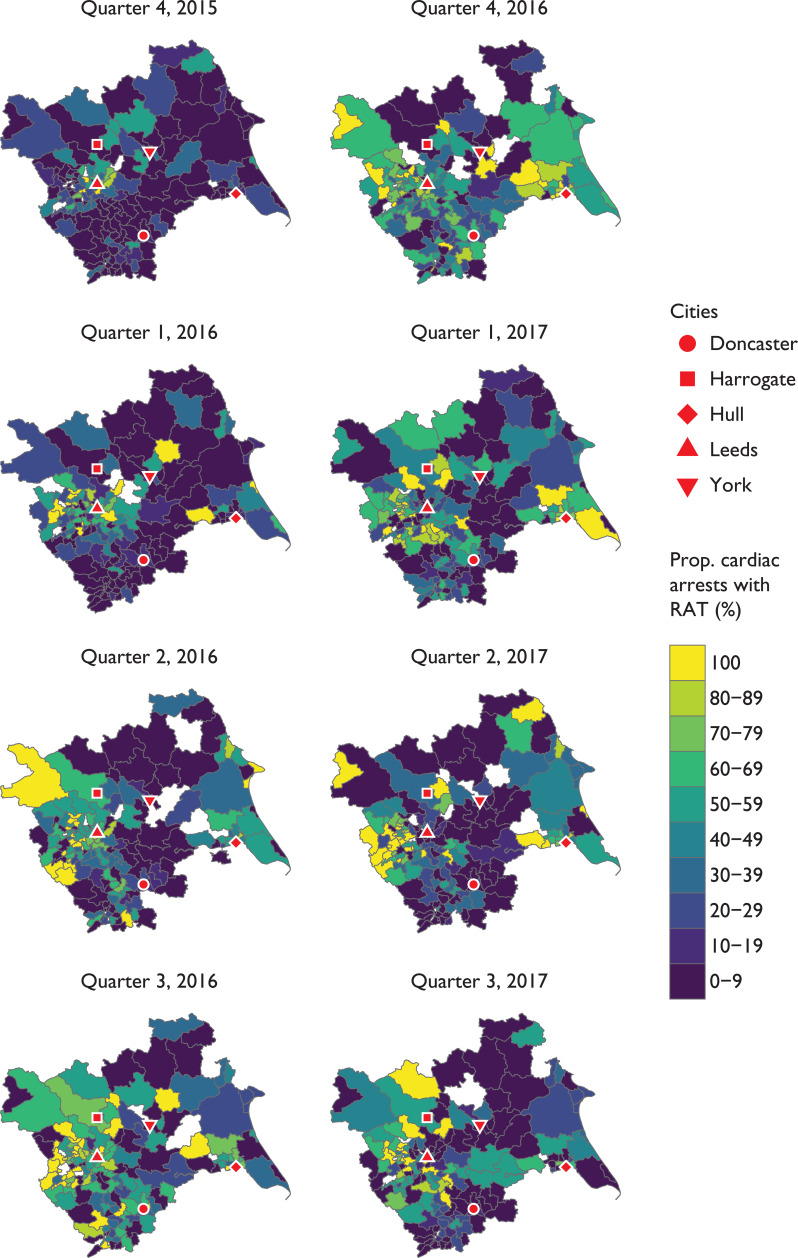
Figure 2. Proportion of cardiac arrests attended by a RAT, stratified by yearly quarter.

The RAT scheme was rolled out across the region over the course of the service evaluation, with coverage expanding from the pilot sites. The proportion of cardiac arrests attended during the data collection period is shown in Figure 2.

### Regression models

The outcome of the regression models for survival to discharge and ROSC on arrival at hospital can be seen in Tables 2 and 3. These results suggest that a RAT on scene is associated with a slight increase in the odds of survival to 30 days (OR 1.01, 95% CI 0.74–1.38, p = 0.93) and odds of ROSC on arrival at hospital (OR 1.13, 95% CI 0.99–1.29, p = 0.08), compared to the odds of not having a RAT present, although neither of these results are statistically significant.

**Table 2.** Results of regression modelling of survival to 30 days.

Variable	OR	95% CI	p-value
Age	0.96	0.95–0.97	0.00
Male gender	1.37	0.99–1.90	0.06
Bystander CPR	0.94	0.65–1.37	0.76
Arrest to crew arrival time (per elapsed minute)	1.00	0.98–1.01	0.62
**Witness status**			
Unwitnessed	Reference	Reference	Reference
Witnessed: bystander	1.78	1.18–2.71	0.01
Witnessed: EMS	4.98	2.55–9.85	0.00
**Presenting rhythm**			
Asystole	Reference	Reference	Reference
PEA	0.95	0.55–1.63	0.85
Shockable	10.11	6.8–15.39	0.00
**Location**			
Private	Reference	Reference	Reference
Public	2.07	1.48–2.88	0.00
Nursing home	0.41	0.11–1.14	0.12
Ambulance	2.07	0.93–4.75	0.08
**Status at hospital**			
ROSC on arrival at hospital	54.82	35.46–89.12	0.00
**RAT**			
No RAT present	Reference	Reference	Reference
RAT present	1.01	0.74–1.38	0.93
RAT present (Unadjusted)*	0.98	0.79–1.21	0.84

*Crude analysis with no adjustments for any other covariates.

**Table 3.** Results of regression modelling of ROSC on arrival at hospital.

Variable	OR	95% CI	p-value
Age	0.99	0.99–0.99	0.00
Male gender	0.83	0.72–0.94	0.01
Bystander CPR	1.00	0.86–1.16	0.95
Arrest to crew arrival time (per elapsed minute)	0.99	0.98–1.00	0.03
**Witness status**			
Unwitnessed	Reference	Reference	Reference
Witnessed: bystander	1.72	1.48–2.00	0.00
Witnessed: EMS	1.88	1.38–2.55	0.00
**Presenting rhythm**			
Asystole	Reference	Reference	Reference
PEA	1.69	1.42–2.00	0.00
Shockable	4.34	3.72–5.08	0.00
**Location**			
Private	Reference	Reference	Reference
Public	1.16	0.98–1.38	0.08
Nursing home	0.96	0.74–1.23	0.74
Ambulance	1.18	0.76–1.82	0.46
**RAT**			
No RAT present	Reference	Reference	Reference
RAT present	1.13	0.99–1.29	0.08
RAT present (Unadjusted)*	1.09	0.97–1.24	0.16

*Crude analysis with no adjustments for any other covariates.

## Discussion

The unadjusted odds ratios suggest that there is no significant increase in the odds of survival to discharge or ROSC on arrival at hospital when a RAT is present, compared to OHCAs where no RAT is present (OR 0.98, 95% CI 0.79–1.21 and OR 1.09, 95% CI 0.97–1.24, respectively). When adjusting for factors that are known to affect outcomes from OHCA using multiple logistic regression, the results from this study indicate that there is a slight increase in the odds of survival to 30 days when a RAT is present (OR 1.01, 95% CI 0.74–1.38, p = 0.93), and in the odds of ROSC on arrival at hospital, compared to OHCA without a RAT present (OR 1.13, 95% CI 0.99–1.29, p = 0.08), although both results are not statistically significant. In addition, a sensitivity analysis was conducted, with variables that did not contribute significantly to the regression model excluded (Supplementary 2). This resulted in no significant difference in the calculation of the odds of survival to discharge or ROSC at hospital when a RAT is present, compared to OHCAs where no RAT is present (OR 1.01, 95% CI 0.74–1.37, p = 0.97 and OR 1.13, 95% CI 0.99–1.29, p = 0.08, respectively).

Drawing firm conclusions about the primary outcome in this study has been impaired by the high level of missing survival outcome data (565/2658, 21.3% of outcomes are missing from the subset of patients who were taken to hospital). In addition, there were some significant differences in the distribution of patient demographic and OHCA factors between the RAT attended and non-RAT attended OHCAs. RAT attended OHCAs had younger patients and a higher proportion of bystander witnessed arrests. Conversely, in the non-RAT OHCA group, there was a significantly higher proportion of witnessed arrests by a member of the ambulance service and cardiac arrests that occurred in an ambulance (Table 1).

It appears from Figure 2 that there was temporal and spatial variation of the proportion of OHCAs attended by a RAT. The scheme rolled out from the pilot sites in October 2015 onwards, and appeared to reach a peak in the third and fourth quarters of 2016. However, the proportion of arrests attended by the RAT declined in 2017. It is possible that this was due to operational pressures resulting in the inappropriate tasking of RAT resources from OHCAs to other emergency calls that could not be covered.

### Comparison with other systems

Making comparisons with the literature is difficult, given that there are limited robust data from other pre-hospital emergency care teams and their effect on survival outcomes. Most published studies compare physician-based critical care teams to ALS paramedics. A recent systematic review found scant evidence that these teams offer a survival benefit in OHCA (von Vopelius-Feldt, Brandling, & Benger, 2017), with three of the six papers included in the review finding no benefit in OHCA outcomes. However, as the authors of the review point out, study design, team tasking and type-2 errors all affect the findings of included studies. It is possible that these teams are of greatest benefit post-ROSC or during protracted resuscitation, if they cannot be dispatched immediately. There is also the suggestion within this systematic review that the attendance of a physician-based critical care team may positively affect the destination hospital, which may have a beneficial impact on patient outcomes.

There are few paramedic-only studies in the UK examining the use of specialist teams to improve outcomes from OHCA. The scheme that has inspired at least two others in England is the 3RU in Scotland. Originally based in Edinburgh, it has now expanded into all urban conurbations. However, the scheme has only published results from early service evaluations, which demonstrated a ROSC rate of 38% in the Edinburgh area in 2010/2011, compared to a national mean of 19.2% at the time (Clegg et al., 2012).

Paramedics forming the 3RU were volunteers who received ALS-style training in addition to non-technical skills (Clarke et al., 2014). However, since the scheme expanded, all training is conducted through paid study leave and staff rostered onto the unit (S. Short, 2018, personal communication).

In the North East Ambulance Service NHS Trust (NEAS), the cardiac arrest response unit (CARU) was set up in 2014 to improve OHCA outcomes. As with the RAT scheme in YAS, it was based on the work of the 3RU. The group was comprised of 11 senior paramedics who provided the majority of coverage, although 11 of the cardiac arrests reported by McClelland, Younger, Haworth, Gospel, and Aitken-Fell (2016) were attended by a pre-hospital emergency medicine (PHEM) doctor who was also a member of the team. Coverage was limited to a single locality focused around Newcastle-Upon-Tyne and working hours of 07:00–17:00. Paramedics forming part of the team completed the pre-hospital emergency resuscitation (SPHERe) course run by Prometheus Medical Ltd. and a pre-hospital anaesthetics course run by the Great North Air Ambulance. Maintenance of skills was achieved by weekly training sessions comprised of ALS drills and scenarios, although most of these were voluntary and attended in the team’s own time.

During its first year of operation, CARU was activated 333 times, and attended 164 OHCAs. Compared to the rest of NEAS, CARU had a significant increase in survival to discharge and ROSC on arrival at hospital (unadjusted odds ratios of 2.08, 95% CI 1.12–3.84 and 1.74, 95% CI 1.19–2.54) (McClelland et al., 2016).

In the London Ambulance Service (LAS), the role of the advanced paramedic practitioner (APP) was created in 2014, and attendance at OHCA is part of the role. The only data published on their performance are from the LAS cardiac arrest report, which shows an increase in ROSC at hospital and survival to discharge figures compared to incidents where no APP was in attendance (34.6% and 12.1% vs. 29.4% and 9.5%). However, as with the previous data, these are unadjusted figures and the report notes that VF/VT was the presenting rhythm in 30.2% of cases attended by an APP compared to 22.0% in other LAS OHCAs (London Ambulance Service NHS Trust, 2017).

### Limitations

This study is observational and retrospective, utilising routine data. As such, causal links cannot be made. In addition, there is a significant proportion of data missing from the primary outcome measure, and the primary outcome is not as patient-centred as survival to discharge with a favourable neurological outcome, for example. However, neurological status of the patient at time of discharge (or to 30 days) is not currently collected as part of the quality indicators for ambulance services. To address issues with data reliability, the Trust is embarking on a roll-out of electronic PCRs, which should improve the reliability of data capture, although it will not guarantee that outcome data will always be provided by receiving hospitals.

No adjustment was made for the receiving hospital in this analysis, which may impact on patient survival outcomes (Stub et al., 2011, 2015). In addition, only a crude adjustment was made to account for the RAT’s alternate role of ceasing futile resuscitation attempts, which may have adversely affected the apparent survival benefit of a RAT presence.

## Conclusion

In this study, the presence of a RAT paramedic was associated with a small increase in survival to 30 days (OR 1.01, p = 0.93) and ROSC on arrival at hospital (OR 1.13, p = 0.08), although neither were statistically significant. The magnitude of missing survival outcomes limits confidence in the robustness of this result. Further research into the effect of roles such as RAT, particularly in schemes lead by paramedics, is required.

## Conflict of interest

RP and DL work for the Yorkshire Ambulance Service NHS Trust. MDT declares no conflict of interest.

## Ethics

Research ethics committee approval was not required as this study was classed as a service evaluation.

## Funding

No sources of funding other than time from respective employers were allocated to the study.

## References

[bibr_1] Clarke S. Lyon R. M. Short S. Crookston C. Clegg G. R. (2014). A specialist, second-tier response to out-of-hospital cardiac arrest: Setting up TOPCAT2. *Emergency Medicine Journal*, 31, 405–407. 23364903 10.1136/emermed-2012-202232

[bibr_2] Clegg G. Sinclair N. Crookston C. Clarke S. Short S. Lyon R. (2012). A program of education, audit and leadership can improve outcomes after out-of-hospital cardiac arrest: The TOPCAT2 projectCategory: Implementation. *Resuscitation*, 83, e1. Retrieved from https://www.resuscitationjournal.com/article/S0300-9572(12)00403-0/fulltext.21798222

[bibr_3] Gräsner J.-T. Lefering R. Koster R. W. Masterson S. Böttiger B. W. Herlitz J. . . . Whittington A. (2016). EuReCa ONE-27 nations, ONE Europe, ONE registry. *Resuscitation*, 105, 188–195.27321577

[bibr_4] London Ambulance Service NHS Trust. (2017). Cardiac arrest annual report 2016/17. Retrieved from https://www.londonambulance.nhs.uk/download/16164/.

[bibr_5] McClelland G. Younger P. Haworth D. Gospel A. Aitken-Fell P. (2016). A service evaluation of a dedicated pre-hospital cardiac arrest response unit in the North East of England. *British Paramedic Journal, * 1(2), 35–41.

[bibr_6] Meaney P. A. Bobrow B. J. Mancini M. E. Christenson J. de Caen A. R. Bhanji F. . . . on behalf of the CPR Quality Summit Investigators, the American Heart Association Emergency Cardiovascular Care Committee, and the Council on Cardiopulmonary, Critical Care, Perioperative and Resuscitation. (2013). Cardiopulmonary resuscitation quality: Improving cardiac resuscitation outcomes both inside and outside the hospital: A consensus statement from the American Heart Association. *Circulation*, 128, 417–435. 23801105 10.1161/CIR.0b013e31829d8654

[bibr_7] NHS England. (2015). Ambulance quality indicators specification guidance. Retrieved from https://www.england.nhs.uk/statistics/wp-content/uploads/sites/2/2013/04/AMB-QI-guidance-v1.4.docx.

[bibr_8] R Core Team. (2017). R: A language and environment for statistical computing. Retrieved from https://www.R-project.org/.

[bibr_9] Stub D. Schmicker R. Anderson M. Callaway C. Daya M. Sayre M. . . . Nichol G. (2015). Hospital post-resuscitative performance is associated with clinical outcomes after out-of-hospital cardiac arrest. *Heart, Lung and Circulation*, 24, s383. 10.1016/j.resuscitation.2015.04.015 25917263

[bibr_10] Stub D. Smith K. Bray J. E. Bernard S. Duffy S. J. Kaye D. M. (2011). Hospital characteristics are associated with patient outcomes following out-of-hospital cardiac arrest. *Heart*, 97, 1489–1494. 21693477 10.1136/hrt.2011.226431

[bibr_11] von Vopelius-Feldt J. Brandling J. Benger J. (2017). Systematic review of the effectiveness of prehospital critical care following out-of-hospital cardiac arrest. *Resuscitation*, 114, 40–46. 28253479 10.1016/j.resuscitation.2017.02.018

